# The extracellular matrix: structure, composition, biological functions, diseases, and therapeutic targets

**DOI:** 10.1186/s43556-026-00436-1

**Published:** 2026-03-26

**Authors:** Khairunnisa Mohd Kamal, Ahmad Rohi Ghazali, Gayathri Thevi Selvarajah, Nurul Syakima Ab Mutalib, Nadiah Abu, Eng Wee Chua, Siti Fathiah Masre

**Affiliations:** 1 Biomedical Science Programme, Centre for Toxicology and Health Risk Studies (CORE), Faculty of Health Sciences, Universiti Kebangsaan Malaysia (UKM), Kuala Lumpur, 50300 Malaysia; 2 Department of Veterinary Clinical Studies, Faculty of Veterinary Medicine, Universiti Putra Malaysia (UPM), Serdang, 43400 Malaysia; 3 Department of Biological Sciences and Biotechnology, Faculty of Science and Technology, Universiti Kebangsaan Malaysia, Bangi, Selangor Malaysia; 4 UKM Medical Molecular Biology Institute, Universiti Kebangsaan Malaysia (UKM), Cheras, Kuala Lumpur, 56000 Malaysia; 5 Centre for Drug and Herbal Development, Faculty of Pharmacy, Universiti Kebangsaan Malaysia (UKM), Kuala Lumpur, 50300 Malaysia

**Keywords:** Extracellular matrix, Epigenetic regulation, DNA methylation, Mechanotransduction, Cancer, Therapeutic targets

## Abstract

The extracellular matrix (ECM) is a highly organised and dynamic regulator of tissue structural integrity and biochemical signalling, and its dysregulation is a hallmark of fibrosis and cancer. Recent evidence highlights the critical role of epigenetic mechanisms in controlling ECM-related gene expression and remodelling activity. This review integrates recent advances in understanding how epigenetic mechanisms govern ECM composition, remodelling, and mechanotransduction, and how reciprocal ECM-derived signals reshape the epigenetic landscape. Growing evidence links DNA methylation, histone modifications, and non-coding RNAs to the regulation of key ECM components, matrix-modifying enzymes, and stiffness-associated signalling pathways, including TGF-β, Wnt, and PI3K/Akt are summarised in this review. The bidirectional feedback between altered ECM mechanics and epigenetic enzyme activity is emphasised, showing how matrix stiffening and aberrant epigenetic programming cooperatively drive pathological tissue remodelling and tumour progression. This review summarises findings from in vitro systems, animal models, and human disease studies that illustrate the functional consequences of ECM-epigenetic crosstalk. The emerging therapeutic approaches targeting the ECM-epigenetic axis, including epigenetic modulators and ECM-directed interventions, outline current challenges and future directions for restoring matrix homeostasis in disease. Together, this review provides an integrated framework for understanding the bidirectional ECM-epigenetic interactions and their translational relevance in molecular biomedicine.

## Introduction

The extracellular matrix (ECM) is a dynamic and highly structured three-dimensional network that supports tissue architecture and maintains organ homeostasis [1, 2], which comprised collagens, elastin, laminins, fibronectin, proteoglycans, and associated macromolecules [2]. The ECM forms an integrated scaffold in which the disruption of individual components can destabilise remodelling processes [3]. Beyond structural support, the ECM transmits biochemical and biomechanical cues that regulate adhesion, migration, proliferation, differentiation, and intracellular signalling, thereby exerting broad control over cellular function and disease behaviour [4, 5]. Concurrently, growing emphasis has been placed on understanding how environmental conditions influence gene expression through epigenetic mechanisms such as DNA methylation, histone modifications, and non-coding RNAs [6, 7]. These reversible processes maintain cellular adaptability and gene regulatory stability [6], and their dysregulation contributes to tumour proliferation, invasion, metastasis, and metabolic rewiring [6, 8–11]. DNA methylation, mediated by DNA methyltransferases (DNMTs), remains one of the most extensively characterised mechanisms, with aberrant methylation patterns altering tumour suppressor and oncogene expression and driving oncogenesis [12, 13].

Mounting evidence reveals a bidirectional crosstalk between ECM dynamics and epigenetic regulation, whereby ECM composition and mechanics modulate the epigenetic landscape, while epigenetic modifications reshape ECM remodelling. This reciprocal relationship suggests opportunities for synergistic therapeutic targeting, yet its broader implications remain insufficiently defined. This review synthesises current understanding of ECM biology, the epigenetic mechanisms governing ECM organisation, how ECM remodelling drives epigenetic reprogramming in cancer and the existing bidirectional crosstalk between ECM and epigenetics in shaping ECM composition. It further examines its dysregulation across common human diseases, including cancer, fibrosis, and cardiovascular and musculoskeletal disorders, and highlights emerging therapeutic strategies, underscoring key research gaps and future directions.

## Basis of the extracellular matrix (ECM)

The ECM is a major constituent of connective tissues, providing the physical maintenance for all cells and is composed of a diverse array of macromolecules, including various proteins, sugars, and other components [14]. The composition of the ECM is not static; rather, it is actively being deposited, remodelled, and degraded, reflecting its dynamic nature and its crucial role in maintaining tissue homeostasis [15], profoundly influencing cell proliferation, differentiation, migration, and wound healing [14]. This inherent dynamism allows the ECM to adapt to changing physiological and pathological conditions, highlighting its importance as an active regulator of cellular and tissue function [16]. Its specific composition and organisation vary greatly between different tissues and organs, dictating their unique mechanical and biochemical properties [17].

**Fig. 1:**
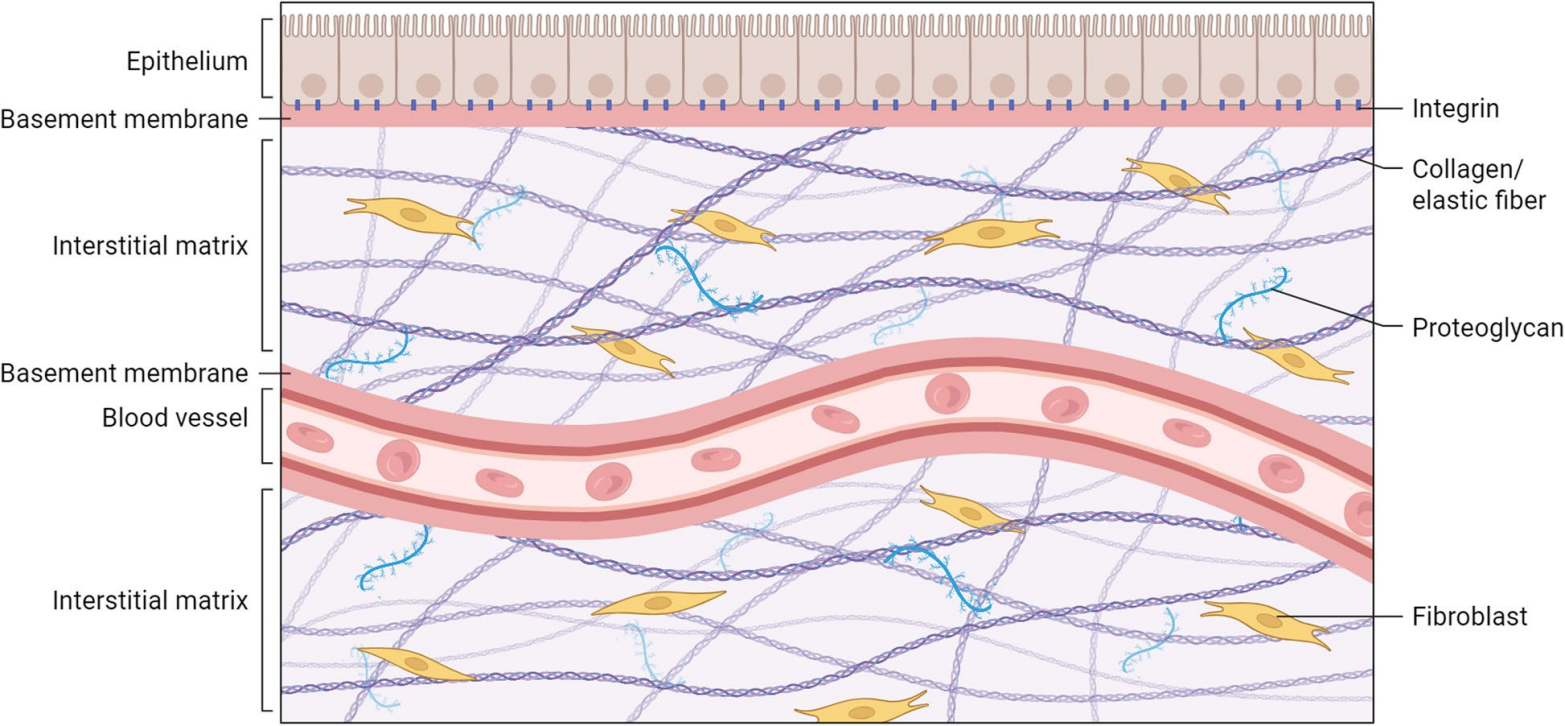
Structural and functional basis of the ECM architecture. The ECM is a hierarchically organised, composite network composed such as fibrillar collagens, elastin fibres, and proteoglycans. Created in BioRender.com

The ECM is mainly classified into two types, based on its localisation and composition: the interstitial matrix (IM) and the basement membrane (BM) [18]. The IM is characterised as a loose network, providing the three-dimensional network found within connective tissues, positioned between cells. It is rich in fibrillar collagens, proteoglycans, fibronectin, and elastin, collectively providing mechanical support and a substrate for cell migration [19] (Fig. 1). Conversely, the BM is a specialised, thin, sheet-like form of ECM that supports epithelial cells and separates them from adjacent stromal cells [20], thereby maintaining the integrity of the structure. It also functions as a microenvironment sensor and as a crucial barrier that separates distinct tissue compartments, primarily composed of type IV collagen and laminins [21]. Beyond its structural roles, the ECM primarily provides structural support and mechanical integrity, offering a physical framework that organises cells into tissues and organs. This framework imparts characteristic shapes and mechanical properties, including elasticity and tensile strength [22], and regulates cell adhesion and migration [23]. Furthermore, the ECM provides binding sites for cell-surface receptors, predominantly integrins, which facilitate cell adhesion, migration, and environmental sensing [24]. As a reservoir for growth factors, cytokines, and other signalling molecules, the ECM also controls their localisation, stability, and presentation to cells, thereby providing essential biochemical cues through specific binding motifs [25]. The ECM also plays a key role in regulating diverse cellular behaviours, influencing proliferation, differentiation, survival, and gene expression by providing both biochemical and physical signals [26]. It further serves as a crucial guide for cell migration, differentiation, and tissue patterning during embryonic development [26]. Ultimately, the ECM plays a crucial role in maintaining tissue homeostasis and repair, particularly in orchestrating the complex processes of wound healing and tissue regeneration following injury [27]. The maintenance process involved complex interactions within the structural components, including collagens, elastin, glycosaminoglycans (GAGs), proteoglycans (PGs), glycoproteins, and key enzymes such as matrix metalloproteinases (MMPs) and lysyl oxidase (LOX), which will be briefly discussed in the next section.

### Structural components

#### Collagens

Collagens are well-known structural proteins in the ECM that provide rigidity and tensile strength to the dynamic, complex framework. It forms triple-helical structures that support diverse types of tissues, like skin, tendons, and bones [28]. Collagens exist in different forms that serve as structural support to various types of tissues, such as Type I, which is commonly found in skin, tendons, ligaments, the softer Type II collagen, mostly in cartilage, and Type III, which is more elastic, such as in blood vessels, muscles, and organs. The different forms contribute to diverse tissue properties ranging from rigidity to flexibility, emphasising their dynamic roles in maintaining tissue health [29, 30].

#### Elastin

Elastin is responsible for elasticity in ECM structures. The elastic fibres are rich in cross-linked elastin, while the outer periphery consists of microfibrils, which enable the tissues to stretch and recoil accordingly [29]. Together with collagen, the balance with elastin is crucial in establishing the mechanical properties of tissues, ultimately influencing their ability to maintain their structural integrity and withstand deformation [29]. In lung ECM, elastin provides the resilience and flexibility required for dynamic functions such as breathing and circulation [29, 31].

#### Glycosaminoglycans (GAGs), proteoglycans (PGs) and glycoproteins

The GAGs are composed of long, linear polysaccharides whose side chains are covalently linked to PGs, forming a water-gel-like matrix that provides hydration, lubrication, and shock absorption, especially in the intervertebral disc and cartilage [32]. GAGs are also responsible for coordinating cell-ECM interactions, which is important in tissue repair and organisation. The involvement of tenascin in cell adhesion, migration and tissue remodelling is highly expressed during development, wound healing and in pathological conditions. Meanwhile, the PGs, located within the intracellular components of the ECM, are characterised by their highly branched structure consisting of protein cores linked to polysaccharides. PGs are predominant in connective tissues and function as organisers of tissue structure, regulating signalling pathways by binding to secreted molecules such as growth factors [28–30, 33, 34]. Glycoproteins, including fibronectin, laminin, and tenascin, are key components of the ECM that support cellular functions, such as cell adhesion, migration, and signalling [29]. These key components of glycoproteins facilitate cell adhesion to the ECM and promote intercellular communication, thereby guiding processes such as cell migration, differentiation, and survival through their interactions with cell-surface receptors [35]. Together, they form a crosslink structure that bridges cells and other ECM components within the tissue matrix [36].

#### Matrix metalloproteinases (MMPs)

Matrix metalloproteinases (MMPs) are a group of zinc- and calcium-dependent endopeptidases that play a central role in ECM degradation and remodelling, targeting a wide range of ECM components [37, 38]. More than 20 MMPs have been identified in humans and are categorised based on their substrate specificity and structural domains into subgroups such as collagenases, gelatinases, stromelysins, and membrane-type MMPs [39]. These enzymes are critically involved in physiological processes like embryonic development, tissue morphogenesis, wound healing, and angiogenesis through their regulation of ECM turnover [40]. The activity of MMPs is tightly regulated by endogenous inhibitors known as tissue inhibitors of metalloproteinases (TIMPs), which not only suppress MMP-mediated proteolysis but also influence cell behaviour, including proliferation, apoptosis, and angiogenesis [41]. A finely tuned balance between MMPs and TIMPs is essential for maintaining tissue homeostasis. Disruption of this balance contributes to various pathological conditions, including fibrosis, chronic inflammation, aberrant angiogenesis, and cancer [42]. In these contexts, MMPs influence cellular adhesion, migration, proliferation, and survival by proteolytically processing bioactive molecules that modulate these critical processes [43].

#### Lysyl oxidase (LOX)

Lysyl oxidase (LOX) is an ECM enzyme that catalyses the covalent crosslinking of collagen and elastin, contributing to tissue structural integrity. Through the oxidative deamination of lysine residues into peptidyl aldehydes, LOX facilitates matrix stabilisation and tensile strength [44, 45], while its catalytic by-products, hydrogen peroxide (H₂O₂) and ammonia, are involved in redox signalling and cellular communication [46]. The enzymes play essential roles in normal growth and development, including connective tissue formation, embryogenesis, and wound healing. It has been found that LOX family enzymes are widely expressed across human tissues, exhibiting distinct spatial and temporal expression patterns that vary with the developmental stage and tissue type [46]. Concurrently, alterations in the tissue microenvironment can disrupt the normal regulation of LOX, leading to aberrant collagen crosslinking and excessive matrix stiffening, which further amplifies ECM dysregulation and promotes disease progression [16, 47].

### Biomechanical and biochemical signalling roles

The ECM is known for its role in providing a physical scaffold that supports tissue architecture, ensuring mechanical stability, and spatial organisation of organs and tissues. Beyond its architectural role, the ECM is a central regulator of tissue homeostasis, orchestrating both biochemical and biomechanical signalling that governs cell fate, differentiation, migration, and survival. Crucially, the ECM is involved in complex signalling pathways, including mechanotransduction, ligand-induced signalling, and growth factor signalling [48], which regulate cellular behaviour and contribute to fundamental biological processes such as growth, development, and ageing. The dynamic nature of the ECM network is continuously synthesised, modified, secreted and assembled [49].

ECM components serve as ligands for various cell surface receptors, notably through ligand-receptor interactions (e.g., integrins, discoidin domain receptors), indirect growth factor modulation (TGF-β, HGF/c-MET), and proteolytic release of bioactive fragments, which regulate gene expression, cell phenotype, and tissue remodelling [50, 51]. Structural components of the ECM interact with integrins, activating intracellular signalling cascades that regulate cell proliferation, differentiation, migration, and survival [52, 53]. Integrins, as primary ECM receptors, play a key role in modulating mechanotransduction pathways by linking the ECM to the intracellular cytoskeleton, thereby enabling cells to sense and respond to their mechanical environment. Mechanical cues from the ECM regulate intracellular tension via integrin engagement, which initiates integrin oligomerisation and conformational changes in adaptor proteins, such as talin [54]. These adaptor proteins integrate mechanosensors, signalling intermediates, actin-binding proteins, and anchor integrins to the cytoskeleton [55].

Its mechanical properties, such as stiffness, elasticity, viscoelasticity and strength, are pivotal in determining cellular behaviour and tissue functionality [29]. The precise mechanical characteristics of the ECM are determined by the intricate interplay and organisation of its molecular components, including collagen, elastin, proteoglycans, glycoproteins, MMPs, and even water content [29]. The stiffness and elasticity of the ECM, often referred to as its "mechanical stiffness", are critical determinants of cell behaviour, influencing processes like proliferation, differentiation, and gene expression [29]. Cells possess the remarkable ability to sense and respond to the mechanical properties of their surrounding environment through sophisticated mechanotransduction mechanisms [29]. Mechanotransduction refers to the ability to translate mechanical signals into biochemical responses through cell interactions, topography, substrate shear flow, and stiffness [55]. It is pivotal to physiological processes and various pathologies, serving as a foundational concept that directly links the ECM's physical state to intracellular gene expression, including epigenetic changes. Intercellular dynamics and ECM rigidity modulate intracellular tension, thereby influencing tissue morphology and stiffness [55]. Within the ECM, adherent junction proteins and focal adhesions are crucial for transmitting multidirectional forces and maintaining structural integrity [56]. These dynamic structures facilitate mechanical stability and cell-microenvironment interactions, converting mechanical forces into biochemical signals by deforming in response to stimuli [20]. Understanding this interconnectedness is vital, as targeting one component might have ripple effects on overall ECM mechanics, which could be therapeutically exploited or lead to unintended consequences.

### Dynamic remodelling in health and diseases

Tissue homeostasis relies on the precise regulation of ECM deposition, modification, degradation, and organisation, which together establish the ECM's biochemical and biophysical properties. Changes in the ECM can significantly impact complex cellular signalling networks [57, 58], as communication between cells & their microenvironment regulates cellular physiology through biophysical signalling [55]. Cells distinguish physical characteristics by generating physical forces through biological mechanisms involving the cytoskeleton and adhesion proteins, which are then distributed between the nucleus and cytoplasm, ultimately producing biological signals that facilitate various signalling pathways and cellular responses [55]. This explains the strict regulations governing ECM components and their microenvironment, which can lead to a series of consequences, including ECM stiffness, through the remodelling mechanism. The ECM changes include various remodelling mechanisms, which can be grouped into four key processes: (1) ECM deposition, which alters the quantity and composition of ECM components, thereby influencing both its biochemical and mechanical properties; (2) post-translational chemical modifications that modify the ECM's biochemical and structural characteristics; (3) proteolytic degradation, which frees bioactive fragments and ECM-bound factors, potentially enabling the release of cellular barriers to migration; and (4) force-mediated physical remodelling, which reorganizes the ECM by aligning fibres and creating pathways for cell migration [3].

In pathological contexts, altered ECM stiffness, particularly increased rigidity, profoundly impacts cell behaviour through enhanced mechanotransduction in both cancer and stromal cells [59]. This is because ECM stiffness is recognised as a prominent biomechanical cue that alters ECM-cell matrix communication and signal transduction within the cellular microenvironment [60]. Dysregulation also leads to other complications, including fibrosis, cardiovascular, and metabolic disorders [28, 50, 53, 61]. Several signalling pathways are intricately mediated by ECM receptors, with specific receptors detecting environmental imbalances that lead to ECM stiffness. This involves the activation of several signalling pathways, such as integrin activation by the stiff matrix, which promotes integrin clustering and enhances downstream signalling [62]. Integrins mediate cell-ECM adhesion, activating intracellular pathways (ERK, FAK, PI3K/AKT) and the FAK/Src pathway [59, 63], which regulate gene expression, proliferation, migration, and differentiation.

The ECM also acts as a reservoir and regulator of bioactive molecule bioavailability and of growth factor (GF) signalling, including TGF-β, VEGF, PDGF, and FGF. The GFs are sequestered within ECM domains and released in response to injury or enzymatic degradation, amplifying local signalling [64]. For instance, TGF-β activation is tightly controlled by integrin-mediated release from latent complexes, driving epithelial-mesenchymal transition (EMT), fibrosis, and cancer progression [50, 65]. Proteolytic remodelling, involving key enzymes such as MMPs and LOXs, modifies ECM structure, which in turn influences angiogenesis, inflammation, migration, and ECM stiffness. ECM degradation and abnormal collagen crosslinking alter the bioavailability of angiogenic and profibrotic factors, contributing to cardiac fibrosis, hypertrophy, and heart failure in cardiovascular diseases [16, 66]. Similarly, disrupted ECM signalling affects the behaviour of chondrocytes and osteoblasts in musculoskeletal disorders, leading to cartilage breakdown and impaired bone remodelling, as seen in osteoarthritis and osteoporosis [67, 68]. Meanwhile, in tumorigenesis, excessive ECM remodelling by MMPs and LOXs alters biochemical signalling, enhancing invasiveness and angiogenesis [53, 69].

## ECM and epigenetic regulation

**Fig. 2:**
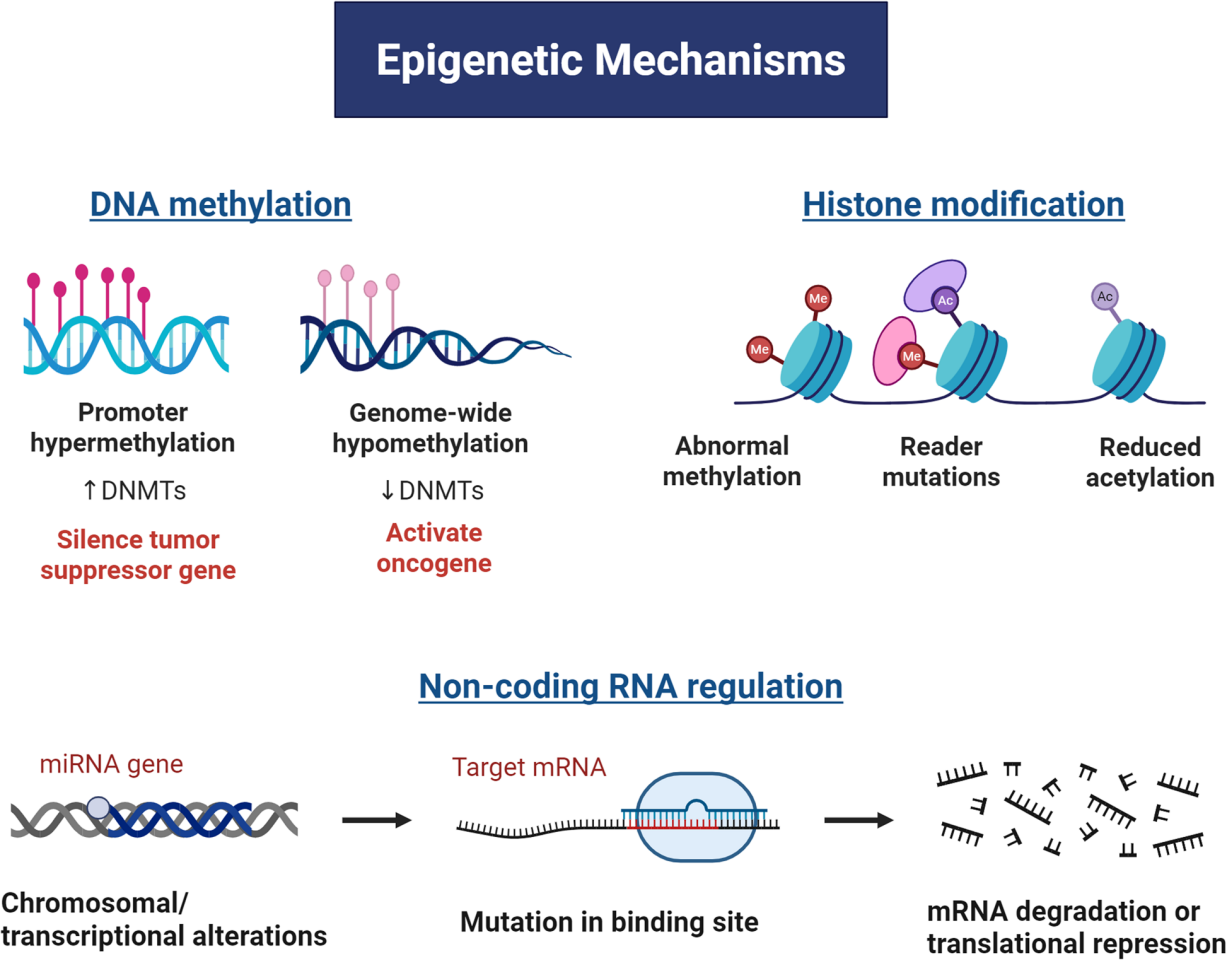
Example of a schematic illustration of the major epigenetic mechanisms in cancer progression. (Left) DNA methylation abnormalities include promoter hypermethylation mediated by increased DNMT activity, leading to tumour suppressor gene silencing, and genome-wide hypomethylation associated with reduced DNMT activity, which may activate oncogenes and promote genomic instability. (Right) Histone modifications encompass aberrant histone methylation, mutations in chromatin reader proteins, and reduced histone acetylation, all of which alter chromatin accessibility and transcriptional activity. (Bottom) Non-coding RNA regulation, particularly miRNAs, is characterised by transcriptional or chromosomal alterations affecting miRNA genes, mutations within miRNA-binding sites on target mRNAs, and subsequent mRNA degradation or translational repression. Together, these interconnected epigenetic mechanisms dynamically regulate gene expression without altering the DNA sequence and contribute to pathological cellular reprogramming across diverse diseases. Created in BioRender.com

Epigenetics plays a key role in the development and maintenance of gene expression in mammalian tissues [9], thereby determining the adaptability of the genotype across different environments [6]. Essentially, these molecular mechanisms influence the transmission of genetic information, resulting in diverse gene expression patterns despite the same DNA sequence [8]. The key mechanisms in epigenetics are DNA methylation, histone modification, nucleosome positioning, and miRNAs, also known as the epigenome [6] (Fig. 2). These mechanisms are crucial for the normal mammalian development and regulation of gene expression, as any disturbance or dysregulation in the mechanism results in improper function of gene regulation, cell growth, and proliferation, which finally leads to diseases such as cancer [6, 9]. In contrast to genetic alterations, epigenetic changes are reversible, offering a promising new avenue for targeted gene therapy aimed at restoring normal gene expression and reversing dysregulated cellular processes [6]. Epigenetic alterations are now recognised as a hallmark of cancer development [12], as the dysregulation of epigenetic mechanisms influences gene alteration and information transmission.

### DNA methylation

DNA methylation refers to the covalent addition of a methyl group to the 5th carbon of cytosine within CpG dinucleotides, resulting in the formation of 5-methylcytosine [70]. This reaction, catalysed by DNA methyltransferases (DNMTs) using S-adenosylmethionine (SAM) as the methyl donor [71], occurs mainly at CpG islands located in gene promoters, which are usually unmethylated under normal conditions [8]. The DNMT family consists of DNMT1, DNMT3A, and DNMT3B, each with distinct roles in regulating DNA methylation [72, 73]. DNMT1 preserves existing methylation patterns during replication [74–76], while DNMT3A and DNMT3B establish de novo methylation during embryogenesis [8, 74, 75, 77]. DNMT3A preferentially methylates CpG sites outside CpG islands [78, 79], while DNMT3B primarily methylates repetitive sequences [80].

DNA methylation stands out as one of the earliest and most extensively studied, given its significant role in altering genomic DNA [8] through gene silencing and chromatin organisation [81], and its association with cancer initiation and progression [12, 13]. Alteration in the methylation pattern could disrupt the normal transcriptional activity at the molecular level, as DNA methylation functions as a ‘switch’ that controls the expression of genes according to the microenvironment. Moreover, epigenetic alterations are more prominent in causing lung cancer than genetic changes, and one key mechanism is DNA methylation [6]. Aberrant DNMT expression during tumorigenesis leads to CpG island hypermethylation and global hypomethylation, thereby disrupting genes involved in DNA repair, cell cycle control, apoptosis, and angiogenesis [8, 9]. Promoter hypermethylation silences tumour expression, whereas genome-wide hypomethylation activates oncogenes and transposable elements, such as LINE-1 and Alu, leading to genomic instability [82–86]. Experimental studies have demonstrated that loss or dysfunction of DNMT significantly impacts development and tumorigenesis in vivo [87–89].

### Histone modification

Histone modifications are covalent chemical changes applied to histone proteins, primarily the N-terminal tails of histones H2A, H2B, H3, and H4, that regulate the accessibility and structural organisation of chromatin [90], determining whether genes are transcriptionally active, poised, or repressed [91]. The epigenetic mechanism involves several key processes, including acetylation, methylation, phosphorylation, and ubiquitination [92], that alter chromatin structure and regulate transcription by modulating the accessibility of genomic regions to the transcriptional machinery [91]. Post-translational modifications are dynamically controlled by writers (HATs, HMTs, kinases, and ubiquitin ligases), erasers (HDACs, demethylases, and phosphatases), and reader enzymes (bromodomains, chromodomains, PHD fingers) that interpret histone marks to recruit transcriptional machinery [93]. ‘Open’ or relaxed chromatin, known as euchromatin, is associated with transcriptional activity, as histone H3 and H4 acetylation and certain methylation marks (e.g. H3K9ac and H3K27ac) increase DNA accessibility and promote gene expression [94]. In contrast, transcriptionally inactive heterochromatin adopts a compact or ‘closed’ configuration in which reduced acetylation and repressive methylation (e.g. H3K27me3) limit access of transcriptional machinery, leading to gene silencing [95]. Histone modifications also serve as key epigenetic regulators for gene expression, cell differentiation, DNA replication, and repair, enabling cells to integrate environmental cues into stable or reversible gene expression programs [96]. Importantly, mechanical and biochemical signals derived from the ECM are now recognised as key upstream regulators of histone modification states.

### Non-coding RNA regulation

Non-coding RNAs (ncRNAs), comprised of microRNAs (miRNAs), long non-coding RNAs (lncRNAs), and circular RNAs (circRNAs) [97] are essential epigenetic regulators of diverse physiological and pathological processes. It is responsible for maintaining the transcriptional and post-transcriptional control of ECM homeostasis and remodelling, by inhibiting the transcription of messenger RNA (mRNA) or binding to proteins [98]. Across fibrosis, cancer, cardiovascular, and musculoskeletal diseases, ncRNA networks interface with ECM stiffness, integrin-mediated mechanotransduction, and TGF-β signalling, forming a dynamic regulatory axis that governs ECM deposition, degradation, and tissue remodelling. ECM stiffness further modifies miRNA expression via mechanotransduction pathways, forming feedback loops that promote fibroblast activation and sustained matrix remodelling. The miRNA family is known to be a major regulator of ECM synthesis and degradation, including miR-29 and miR-21. The former acts as an anti-fibrotic agent, suppressing the expression of fibronectin and collagen genes, which are consistently downregulated in fibrotic tissues, thereby contributing to ECM deposition [99]. Meanwhile, the latter is a pro-fibrotic miRNA which promotes TGF-β-driven fibroblast activation and matrix deposition [100].

The architecture of ECM is also influenced by lncRNAs, which modulate chromatin modifications, transcriptional control, and interactions with ECM-related signalling pathways. For example, the lncRNA HOTAIR is responsible for recruiting the PCR2 complex, including EZH2, for chromatin reprogramming and promoting pro-fibrotic ECM gene expression [101], while H19 induced fibroblast-to-myofibroblast differentiation and collagen production via TGF-β/SMAD signalling [102]. For circRNA, it mainly acts as a miRNA sponge through the competitive endogenous RNA network, sequestering miRNAs and upregulating target genes. For instance, circHIPK3 promotes collagen accumulation by sequestering anti-fibrotic miRNAs that bind to miRNA [103], which hampers the effect and reduces their availability to the target messenger RNA (mRNA). The binding of the circRNA inhibits the miRNA repressive effects, thereby indirectly upregulating the expression of target mRNA and corresponding proteins, such as collagen proteins or matrix-remodelling enzymes [104].

### ECM-driven epigenetic reprogramming in cancer

Epigenetic alterations are now recognised as central contributors to tumour initiation, progression, immune evasion, angiogenesis, metastasis, and clinical outcome [105, 106]. As tumour heterogeneity masks cell-specific changes, analysing distinct subpopulations is crucial for identifying cells most affected by aberrant methylation [107]. Typically, dysregulated methylation silences genes, disrupts cell–ECM adhesion, and enhances metastatic behaviours. DNA methylation plays a major role in shaping the TME, particularly by modulating ECM components, ECM-associated genes, and signalling pathways that drive cancer progression. Notable examples include hypermethylation-driven repression of integrin α4 in gastric cancer [108], TIMP-3 in oral cancer [109], FBN2 and IGFB2 in colon cancer [110], and FBLN2 in NSCLC [111]. Collagen genes are also affected, such as COL7A1 hypermethylation, which correlates with poor outcomes in mammary and prostate cancers [112, 113]. Methylation additionally influences cellular metabolism, where elevated one-carbon metabolites upon ECM detachment correlate with hypermethylation [114]. Prognostically, methylation signatures such as hypomethylation-induced VTCN1 overexpression in ovarian cancer predict poor survival and immune infiltration [115]. Therapeutically, DNMT inhibitors exhibit context-dependent outcomes, as they may enhance metastasis by reactivating MMPs through PI3K signalling [116] or, conversely, suppress proliferation by inhibiting collagen and Wnt/β-catenin pathway activation [117].

ECM cues remodel chromatin through extensive histone modification changes that regulate proliferation, motility, and metastasis [118–121], creating a feed-forward loop that sustains oncogenic ECM dynamics [122]. In CAFs, elevated HDAC activity, such as HDAC6, which induces histone hypoacetylation and represses tumour-suppressive programs, including reduced H3K9Ac at the integrin α4 promoter in gastric cancer [108]. Conversely, hyperacetylation can activate metastatic pathways. In HCC, reduced ACOT12 elevates acetyl-CoA levels, thereby increasing H3K9/H3K56 acetylation and driving pro-metastatic gene expression [123]. ECM-regulated shifts in histone methylation also promote tumour progression: reduced EZH2 and H3K27me3 support lung-cancer growth and immune escape [124], while SetD7-dependent H3K4 activation enhances antioxidant signalling in prostate cancer [125]. In CAFs, loss of H3K27me3 and H3K4me3 disrupts integrin α4 regulation and promotes metastasis [122, 126]. ECM stiffness further influences chromatin states by modulating the activity of demethylases and mechanotransduction pathways. High-rigidity matrices upregulate KDM6B via YAP/TAZ, decreasing H3K27me3 and inducing EMT transcription in thyroid carcinoma [127, 128]. In contrast, stiffness-driven YAP activation in HCC enhances stemness and broader chromatin remodelling [129]. Increased matrix rigidity also elevates global H3K27ac and H3K4me3, reinforcing proliferation, stemness, and immune evasion [130], and collagen-dense microenvironments strengthen EMT-associated histone signatures [131]. Mechanotransduction via integrins, FAK, and YAP/TAZ stimulates HDAC3/8 activity in breast epithelial cells, promoting tumorigenic chromatin configurations [132]. Additionally, combined TGF-β1 signalling and stiffness increase EZH2 nuclear localisation and H3K27me3, stabilising EMT programs [133].

The ncRNAs act as key epigenetic regulators, reinforcing ECM-driven tumour progression. ECM stiffness, altered collagen architecture, and integrin mechanotransduction modulate upstream regulators of miRNAs, lncRNAs, and circRNAs through YAP/TAZ, PI3K/AKT, and TGF-β pathways [134, 135]. Anti-fibrotic miRNAs such as miR-29, a major collagen suppressor, are commonly reduced in cancer, enabling excessive ECM deposition and invasion [136]. Conversely, miR-21 is upregulated by stiff ECM and TGF-β, promoting fibroblast activation, immunosuppression, and matrix accumulation [137]. miRNA-containing exosomes released by CAFs further enhance invasion, as seen in oral SCC [138, 139]. lncRNAs also regulate chromatin modification, such as the HOTAIR, which recruits PRC2 to deposit H3K27me3 at tumour-suppressor loci and fosters metastasis and stromal reprogramming [101], while H19 strengthens pro-fibrotic TGF-β/SMAD signalling and collagen synthesis [140]. CircRNAs such as circHIPK3 promote tumorigenesis by inhibiting anti-invasive miRNAs and enhancing ECM-driven migration [103, 141]. Collectively, these ECM-epigenetic circuits reinforce stable epigenetic programs that drive cancer plasticity, metastasis, and resistance (Table 1).

**Table 1:** Examples of ECM-driven reprogramming in cancer involving epigenetic mechanisms

Epigenetic Mechanism	ECM Influence	Key Outcomes in Cancer	Examples
DNA Methylation	ECM stiffness, altered adhesion, metabolic changes	Gene silencing, EMT, metastasis, prognosis	Integrin α4 [108]; TIMP-3 [109]; FBN2/IGFB2 [110]; FBLN2 [111]; COL7A1 [112, 113]; VTCN1 [115]
Histone Acetylation/Deacetylation	HDAC activation in CAFs; collagen-rich matrices; integrin–FAK–YAP/TAZ signalling	TSG repression, oncogene activation, EMT, and immune evasion	HDAC6/CAF hypoacetylation [108]; ACOT12 loss in HCC leads to H3K56/H3K9 hyperacetylation [123]; HDAC3/8 activation in stiff matrices [132]
Histone Methylation/Demethylation	ECM stiffness modulates methyltransferases and demethylases	Loss of repressive marks, EMT gene activation, and stemness	Downregulation of EZH2 caused low expression of H3K27me3 in lung cancer [124]; Knockdown of Setd7 reduced H3K4 in prostate [125]; KDM6B via YAP/TAZ [127, 128]
Chromatin State Changes	Stiff ECM; collagen density	Increased active marks (H3K27ac, H3K4me3), tumourigenic chromatin accessibility	Global H3K27ac/H3K4me3 increased [142]; EMT-linked marks [131]
miRNAs	Matrix stiffness, integrin signalling, TGF-β	Collagen regulation, fibroblast activation, invasion	Downregulation of miR-29 observed in anti-fibrotic [136]; Upregulation of miR-21 as pro-oncogenic [137]; EV-mediated miRNAs [138, 139]
lncRNAs	ECM remodelling, YAP/TGF-β pathways	Chromatin modification, EMT, fibrotic ECM	Overexpression of HOTAIR increased the localisation of PRC2/H3K27me3 [101]; Overexpression of H19 induced TGF-β/collagen expression [140]
circRNAs	ECM stiffness, migration machinery	miRNA sponging, migration, and ECM-driven tumorigenesis	circHIPK3 promotes invasion [103, 141]

### Bidirectional crosstalk: how epigenetics shapes ECM composition

The ECM is now recognised as a powerful regulator of the epigenetic landscape in both physiological and pathological contexts. ECM-derived biochemical cues and mechanical properties function as upstream modulators of DNA methylation, histone modifications, and ncRNA networks, collectively shaping long-term gene expression programs across cancers, fibrosis, cardiovascular disease, musculoskeletal degeneration, neurological disorder and autoimmune diseases. Notably, ECM stiffness is a critical determinant of epigenetic reprogramming. Mechanical forces transmitted through integrins, cytoskeletal tension, and nuclear deformation modulate the activity, expression, and nuclear localisation of DNMTs, driving both global and locus-specific methylation changes. It is observed that vascular smooth muscle cells exposed to stiff microenvironments undergo DNMT1-mediated hypermethylation and heightened inflammatory gene expression, demonstrating that ECM mechanics directly influence methylation-dependent transcriptional states [143]. Similarly, mouse embryonic fibroblasts cultured on rigid substrates exhibit global DNA hypermethylation via PKCα-dependent nuclear import of DNMT3L, revealing a conserved mechanosensitive response across cell types [144].

Importantly, ECM stiffening does not uniformly increase methylation. Endothelial cells grown on rigid matrices display reduced 5-methylcytosine levels relative to soft substrates, suggesting that methylation dynamics are cell-type specific and context dependent [145]. In bladder smooth muscle cells, matrix alterations regulate DNMT3A localisation and activity, linking ECM remodelling to phenotypic switching and lineage stability [146]. In malignancy, ECM stiffening synergises with tumour-associated matrix remodelling to reconfigure DNA methylation landscapes. In gastric cancer, stiff microenvironments promote oncogenic YAP activation through TET2-KMT2A-dependent methylation at the YAP promoter, providing a mechanochemical route for tumour progression [147]. Stem cell systems demonstrate parallel behaviour: human periodontal ligament stem cells grown on stiff matrices display increased global methylation and elevated DNMT3A/3B expression, enhancing osteogenic differentiation [148]. A similar mechanochemical-epigenetic loop drives fibrosis development. Persistent matrix stiffening not only activates fibroblasts but also sustains methylation of fibrogenic genes by altering metabolite availability, including S-adenosylmethionine (SAM), thereby modulating DNMT and TET activity [149, 150]. This contributes to a long-lasting cellular “memory” of injury, perpetuating fibrosis even after the initiating insult resolves.

ECM signals also exert profound control over histone modifications. Mechanical cues, such as matrix stiffness, activate integrin-dependent mechanotransduction pathways, including YAP/TAZ, MRTF-A, and RhoA/ROCK, which converge on chromatin-modifying enzymes to regulate histone acetylation and methylation. Stiff microenvironments elevate H3K9ac and H3K27ac through enhanced HAT activity, driven by increased actomyosin tension and nuclear mechanotransduction, which promotes transcription of adhesion, proliferation, and pro-survival genes [151]. In fibroblasts, ECM stiffness increases H3K9me3 and H3K36me3, stabilising myofibroblast differentiation and reinforcing profibrotic gene expression programs [152]. Conversely, soft matrices suppress acetylation and promote deposition of repressive marks such as H3K27me3 through enhanced PRC2 recruitment [151]. ECM composition further influences histone dynamics. Collagen-rich or fibronectin-rich matrices activate integrin-FAK-ERK/PI3K signalling, altering localisation and activity of HATs and HDACs to modulate chromatin accessibility [153]. Histone-mediated chromatin remodelling promotes cardiac fibrosis, cartilage degeneration, liver and lung fibrosis, and tumour progression through lineage reprogramming, enhanced proliferation, EMT induction, and immune evasion.

Epigenetic regulation mediated by ncRNA is strongly influenced by the mechanical and biochemical properties of the ECM. It is observed that matrix stiffness and TGF-β signalling can suppress miR-29 expression, a key negative regulator of collagen and fibronectin synthesis; reduced miR-29 is implicated in fibrosis across multiple tissues [99, 154]. In parallel, miR-21 is upregulated by TGF-β in fibroblasts and promotes their activation and transformation into myofibroblasts, contributing to fibrosis and ECM deposition via targeting genes such as Jagged1 [100]. lncRNAs also integrate ECM-derived signals such as H19, which is upregulated in fibrotic contexts and suppresses miR-29a/b, leading to increased VEGFA and TGF-β-driven collagen expression in cardiac fibroblasts [155]. These changes may reinforce ECM remodelling at the chromatin level, as dysregulation of miRNAs and lncRNAs by matrix stiffening contributes to tumour invasiveness, EMT, and treatment resistance. From the complex, interconnected linkage between ECM and epigenetic factors, it is critically shown that the interplay has broad implications across organ systems (Table 2).

**Table 2:** The bidirectional crosstalk between epigenetics and ECM composition

Category	Key Findings	Cell/Disease Context	Epigenetic Mechanism	Refs.
DNA Methylation	Stiff ECM induces DNMT1-dependent hypermethylation and inflammatory gene expression	Vascular smooth muscle cells	Induces DNMT1 activity; promoter hypermethylation	[143]
	Stiff substrates trigger PKCα-dependent nuclear import of DNMT3L, causing global hypermethylation	Mouse embryonic fibroblasts	Nuclear DNMT3L localisation; global methylation increase	[144]
	Rigid ECM reduces global 5mC levels; methylation response is cell-type specific	Endothelial cells	Reduced 5mC; altered DNMT/TET activity	[145]
	Matrix remodelling alters DNMT3A localisation and activity	Bladder smooth muscle cells	DNMT3A redistribution; phenotypic switching	[146]
	ECM stiffening reprograms methylation at YAP promoter via TET2-KMT2A	Gastric cancer	Locus-specific methylation; YAP activation	[147]
	Stiff ECM increases DNMT3A/3B expression and global methylation, promoting osteogenesis	Human periodontal ligament stem cells	Induces DNMT3A/3B; global hypermethylation	[148]
	Persistent stiffness alters SAM availability, sustaining fibrogenic methylation	Fibrosis (lung/liver/kidney)	Metabolic–epigenetic regulation of DNMT/TET	[149, 150]
Histone Modifications	Matrix stiffness activates YAP/TAZ, MRTF-A, RhoA/ROCK to increase H3K9ac and H3K27ac	Multiple cell types under mechanical stress	Increase HAT activity; acetylation-mediated transcription	[151]
	Stiff ECM induces H3K9me3 and H3K36me3 to stabilise myofibroblast differentiation	Fibroblasts	Repressive/pro-stability histone methylation	[152]
	Soft matrices enhance PRC2 recruitment, increasing H3K27me3	Cells on compliant substrates	Increase H3K27me3; transcriptional repression	[151]
	Collagen/fibronectin-rich ECM activates FAK–ERK/PI3K to modulate HAT/HDAC localisation	ECM-rich tumour and fibrotic tissue	Altered chromatin accessibility; histone regulation	[153]
ncRNA Regulation	Stiffness and TGF-β suppress miR-29, promoting collagen/fibronectin deposition	Fibrosis (multi-organ)	Downregulate miR-29; pro-fibrotic ECM gene expression	[99, 154]
	miR-21 upregulated by TGF-β; promotes fibroblast activation via Jagged1 targeting	Fibroblasts; fibrotic disease	Upregulate miR-21; myofibroblast transformation	[100]
	H19 upregulated in fibrosis; suppresses miR-29a/b, increasing VEGFA and collagen	Cardiac fibroblasts	lncRNA-miRNA axis regulating ECM genes	[155]

## ECM dysregulation in human diseases

**Fig. 3:**
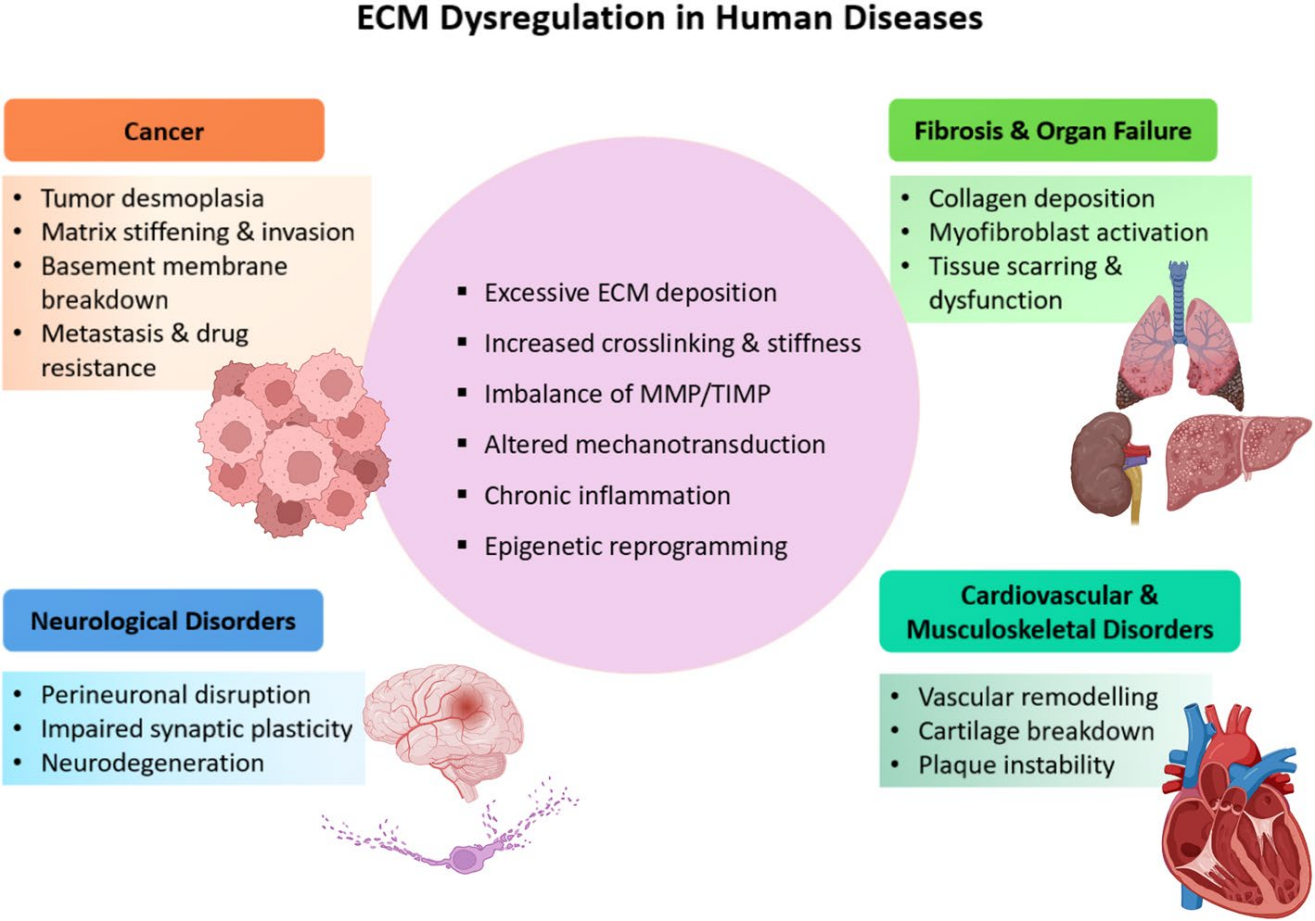
Common hallmarks of ECM dysregulation across human diseases. Aberrant ECM deposition, excessive crosslinking, dysregulated proteolysis, altered mechanotransduction, inflammatory signalling, and epigenetic reprogramming represent shared mechanisms underlying ECM pathology. These conserved processes manifest in distinct disease contexts, including cancer, fibrosis, cardiovascular and musculoskeletal disorders, neurological diseases, and autoimmune conditions. Created in BioRender.com

The cellular microenvironment, shaped by cell–cell and cell-ECM interactions as well as growth factor signalling, is essential for maintaining cell identity and responsiveness to external cues [156]. In healthy tissue, ECM remodelling is a tightly regulated process critical for development, regeneration, and homeostasis, mediated by MMPs and their inhibitors, TIMPs [41, 157]. Increasing evidence highlights the microenvironment’s role in disease pathogenesis, particularly how ECM composition and stiffness influence tissue architecture, polarity, and proliferation [156], such as commonly seen in cancers, fibrosis, cardiovascular, metabolic, neurological, and autoimmune diseases (Fig. 3).

### Cancers

**Fig. 4:**
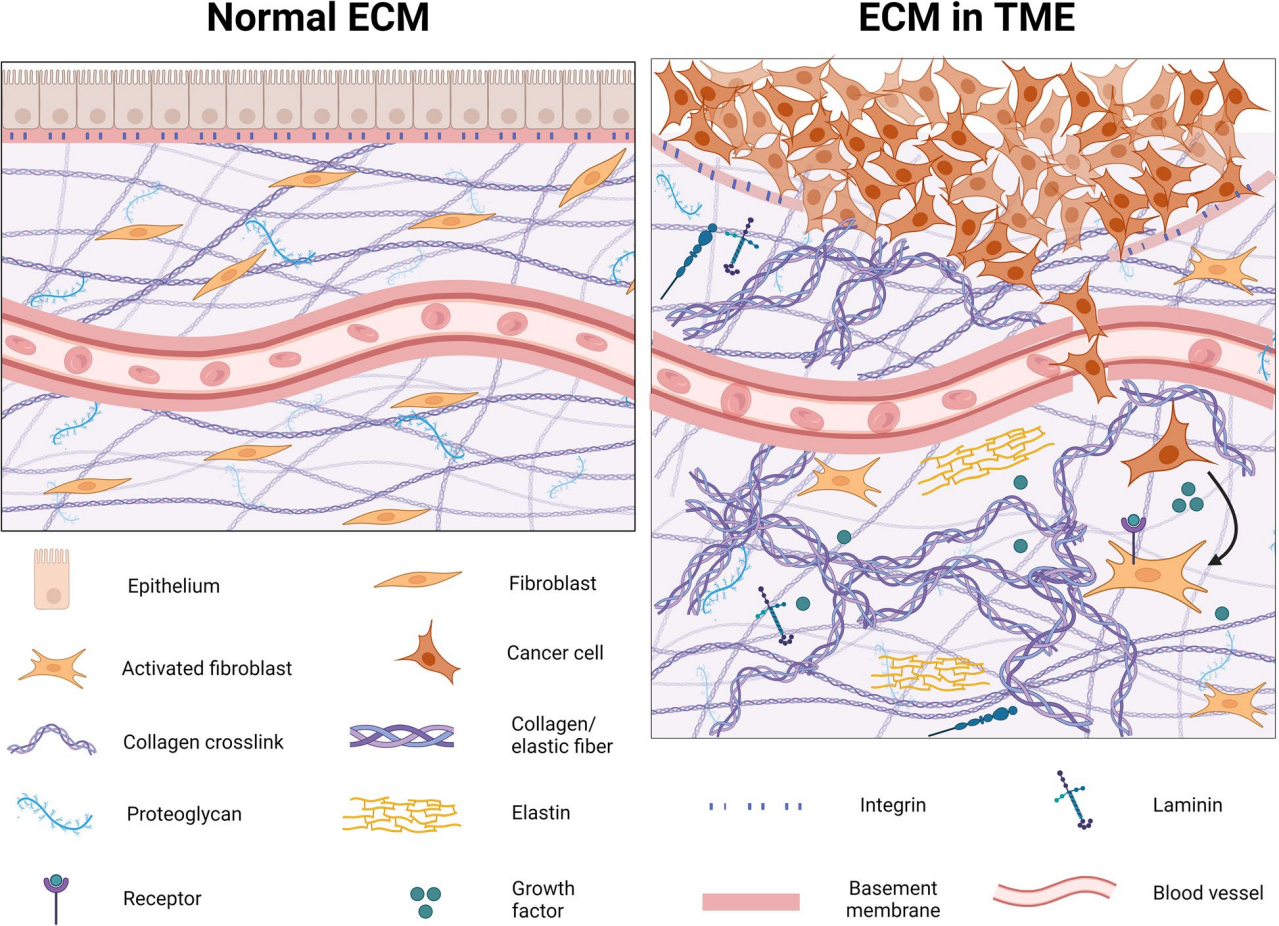
Schematic illustration of ECM dysregulation in TME, leading to cancer progression. The normal ECM (left) maintains well-regulated ECM homeostasis; meanwhile, dysregulation of ECM architecture facilitates CAF migration and disrupts the balanced regulation of ECM structural components. Created in BioRender.com

Cancer remains one of the leading causes of mortality worldwide and is strongly influenced by the tumour microenvironment (TME), an enclosed niche that supports tumour development and progression. The TME functions as a reservoir composed of a complex aggregation of cellular components, including tumour cells, immune cells and the ECM components [158]. Extensive research over recent decades has shown that the TME critically regulates tumour growth, progression, and immune escape by altering the behaviour and interactions of surrounding cellular and non-cellular elements, with the expression and interplay of each component contributing to the fate of tumour progression [159]. Within this microenvironment, the ECM undergoes significant structural and compositional alterations, as the tightly regulated system becomes disrupted during tumorigenesis, collectively termed ECM dysregulation, which profoundly increases matrix stiffness and influences cancer initiation (Fig. 4). The aberrant remodelling further drives tumour proliferation, invasion, metastasis, and therapy resistance through altered mechanotransduction pathways [160–163], by providing physical tracks and biochemical cues for the dissemination of cancer cells [49]. Increased ECM stiffness, primarily arising from excessive collagen deposition and crosslinking, generates a bioactive microenvironment that further promotes tumour growth, immune evasion, and angiogenesis [162–165].

Cancer-associated fibroblasts (CAFs) and tumour-associated macrophages (TAMs), are the main key players which remodel the ECM by secreting TGF-β, collagen, fibronectin, and proteases, resulting in a stiffer, desmoplastic stroma that enhances EMT, invasion, and immunosuppression [28, 33, 166–179]. Enzymes such as MMPs and LOXs further modify ECM architecture, facilitating tumour invasion and angiogenesis [180–188]. The release of ECM fragments by MMP activity can serve as potential biomarkers of tumour invasiveness [189–193]. ECM components such as fibronectin, laminin, collagen, and elastin act not only as structural scaffolds but also as signalling modulators. Fibronectin and laminin are frequently upregulated in cancers, influencing proliferation, migration, and survival, while correlating with poor prognosis [2, 4, 194–198]. Overexpression of glycoproteins such as thrombospondin-1/2 has also been observed in squamous cell carcinoma (SCC), with strong correlations to other ECM elements that may alter matrix organisation and tumour behaviour [199, 200]. Moreover, ECM networks function as interconnected macromolecular systems whose collective regulation remains an emerging area of study [201].

The ECM dysregulation also impacts the immune surveillance. Aberrant collagen organisation mediated by COL11A1, CTHRC1, and COL10A1 has been implicated in T cell exclusion and immune evasion, suggesting that CAF-derived ECM signatures can influence responses to immune checkpoint therapy [202–205]. Mechanistically, ECM-cell interactions are largely transduced through integrins, which activate the FAK/Src pathway upon ligand binding to regulate adhesion, motility, and oncogenic signalling [135, 206–209]. Importantly, the expression of integrins and integrin-linked kinases is also subject to epigenetic regulation, linking ECM signalling with chromatin-level control of gene expression [4, 209]. Overall, ECM dysregulation sustains a feed-forward loop of biochemical and biomechanical signalling that reinforces tumour progression, invasion, and therapeutic resistance.

### Fibrosis and organ dysfunction

In normal tissues, the composition and turnover of the ECM are tightly regulated through a coordinated balance of matrix synthesis, degradation, and remodelling. Disruption of this homeostatic equilibrium, such as during chronic injury or persistent inflammation, often shifts the balance toward excessive deposition of matrix components, particularly collagens and fibronectin, which, in turn, generates a progressively stiffer microenvironment [210]. Fibrosis represents a pathological state in which irreversible, excessive ECM accumulation leads to tissue scarring, architectural distortion, and ultimately organ failure [211, 212]. Aberrant ECM remodelling also perturbs key cellular signalling pathways, especially those mediated by integrins and other mechanosensitive receptors that govern cell differentiation, migration, and overall tissue homeostasis [213]. Remodelling-associated biochemical alterations directly affect cell behaviour and are characteristic of many fibrodegenerative diseases [213, 214]. Sustained hyperglycaemia, for example, activates metabolic pathways that enhance ECM deposition and structural remodelling, promoting fibrosis in multiple organ systems [215]. Several molecular signalling cascades underpin these processes. TGF-β, a principal regulator of ECM synthesis, promotes fibroblast proliferation and myofibroblast activation, thereby accelerating matrix production [216]. Conversely, dysregulated MMP activity impairs controlled ECM degradation, contributing to aberrant accumulation and defective tissue repair [217]. These biomechanical changes also propagate chronic inflammation, oxidative stress, and ongoing tissue injury [215, 218]. The resulting fibrotic remodelling is a major contributor to dysfunction in chronic liver disease [219], kidney [220], and lung diseases [221]. Collectively, persistent matrix deposition, increased collagen cross-linking, and progressive stiffening disrupt normal tissue architecture and impair organ function [222]. ECM dysregulation is therefore a central driver of fibrosis and progressive organ dysfunction.

### Cardiovascular and musculoskeletal disorders

In the cardiac microenvironment, multiple non-myocyte cell types populate the interstitium and actively regulate ECM dynamics [223]. The epicardium, which covers the heart surface, is derived from mesothelial proepicardial cells, which then differentiate into fibroblasts, endothelial cells, and smooth muscle cells [224]. These cells produce and release most matrix proteins, and cell-ECM communication plays an essential role in the programming, homeostasis, and development of heart function and cardiac morphogenesis. Fibroblasts, the predominant ECM-producing cells, maintain myocardial structure and mediate cell signalling through integrins and growth factor interactions [225]. Mesenchymal stromal cells also deposit ECM components such as fibronectin and collagen to promote cellular adhesion and organisation [226], while endothelial cells contribute to vascularisation and remodelling via the synthesis of collagens, laminin, elastin, proteoglycans, and MMPs [227]. Immune cells in the cardiac niche release MMPs that regulate inflammation and cardiomyocyte survival [228]. The cardiac ECM not only supports tissue but also modulates cell motility, survival, and proliferation by acting as a signal transducer for cell–cell communication. Additionally, the ECM transfers mechanical stresses throughout the organ [229] and controls other molecules in the interstitial space [230]. Through myocyte alignment, modulation of blood flow during contraction, compliance, and preservation of the proper tissue tensile modulus, the ECM is also necessary for effective cardiac function [231]. Consequently, the ECM is essential for preserving proper cardiac integrity and pump function [229]. ECM homeostasis depends on a tight balance between MMPs and TIMPs, which collectively regulate ECM components during cardiac remodelling [232–234]. Therefore, disruption of the ECM homeostasis caused cardiac damage, which concurrently occurred with cardiac dysfunction, pathologic remodelling, and fibrosis. Collectively, these cell-ECM interactions orchestrate cardiovascular development and adaptation, with ECM imbalance contributing to the development of fibrosis, hypertrophy, and heart failure.

In musculoskeletal tissues, the ECM provides both structural and regulatory frameworks that maintain the integrity of bone, cartilage, tendons, and muscles. Chondrocytes in cartilage secrete type II collagen and aggrecan, thereby maintaining the compressive resilience of cartilage, while osteoblasts and osteocytes produce type I collagen and osteocalcin to support mineral deposition [67, 68]. Dysregulated ECM turnover, mediated by altered MMP/TIMP activity, leads to tissue degradation and pathologies such as osteoarthritis and osteoporosis [235]. Fibroblasts in tendons and ligaments synthesise fibrillar collagens and tenascins to sustain tensile strength, but excessive mechanotransduction and profibrotic signalling following overuse can trigger fibrotic remodelling [16]. ECM stiffness also modulates stem cell fate, with stiff matrices promoting osteogenic differentiation and softer ones favouring chondrogenic or myogenic lineages [134]. Together, ECM-mediated biochemical and biomechanical signalling governs organ-specific cell behaviour and homeostasis. Aberrant ECM remodelling thus represents a connected pathological mechanism across cardiovascular and musculoskeletal systems, offering a promising avenue for targeted regenerative and anti-fibrotic therapies.

### Other diseases

ECM remodelling plays a critical role in the pathogenesis of tissue inflammation and metabolic dysfunction, including obesity, type 2 diabetes mellitus (T2DM), and non-alcoholic fatty liver disease (NAFLD). Excessive deposition of collagens, fibronectin, and proteoglycans in adipose tissue increases matrix stiffness, which impairs adipocyte expandability, promotes hypoxia, and triggers chronic low-grade inflammation [52, 236, 237]. ECM stiffening also alters integrin-mediated signalling and mechanotransduction pathways, leading to insulin resistance in adipocytes and hepatocytes [238]. Progressive ECM accumulation in the liver, driven by activated hepatic stellate cells, underlies the transition from steatosis to steatohepatitis and fibrosis, highlighting the critical impact of ECM dysregulation in metabolic disease severity [239, 240].

The ECM is essential for synaptic plasticity, neuronal migration, and network stability in the nervous system. Alterations in brain ECM components, particularly perineuronal nets (PNNs), are increasingly implicated in neurodegenerative and neuropsychiatric disorders; meanwhile, abnormal ECM remodelling contributes to impaired synaptic plasticity, neuroinflammation, and amyloid-β deposition in Alzheimer’s disease [241, 242]. Additionally, dysregulated expression of chondroitin sulfate proteoglycans restricts neural regeneration and exacerbates neurodegeneration following injury [243]. Similarly, ECM stiffness and altered glycoprotein composition influence glial activation and mechanosensitive signalling, contributing to disease progression in multiple sclerosis and Parkinson’s disease [244, 245].

Alterations in ECM architecture also contribute to autoimmune diseases by shaping immune cell activation, migration, and persistence within inflamed tissues. This is because the altered collagen architecture and increased matrix stiffness enhance fibroblast-like synoviocyte activation, sustaining chronic inflammation. In rheumatoid arthritis, excessive ECM degradation and remodelling in the synovium promote immune cell infiltration, angiogenesis, and joint destruction [246]. In systemic sclerosis, excessive ECM deposition driven by activated fibroblasts leads to widespread tissue fibrosis and vascular dysfunction [116]. Similarly, in inflammatory bowel disease, disrupted ECM organisation alters epithelial barrier integrity and immune-stromal crosstalk, perpetuating chronic intestinal inflammation [247].

## Therapeutic targeting of the ECM

**Fig. 5:**
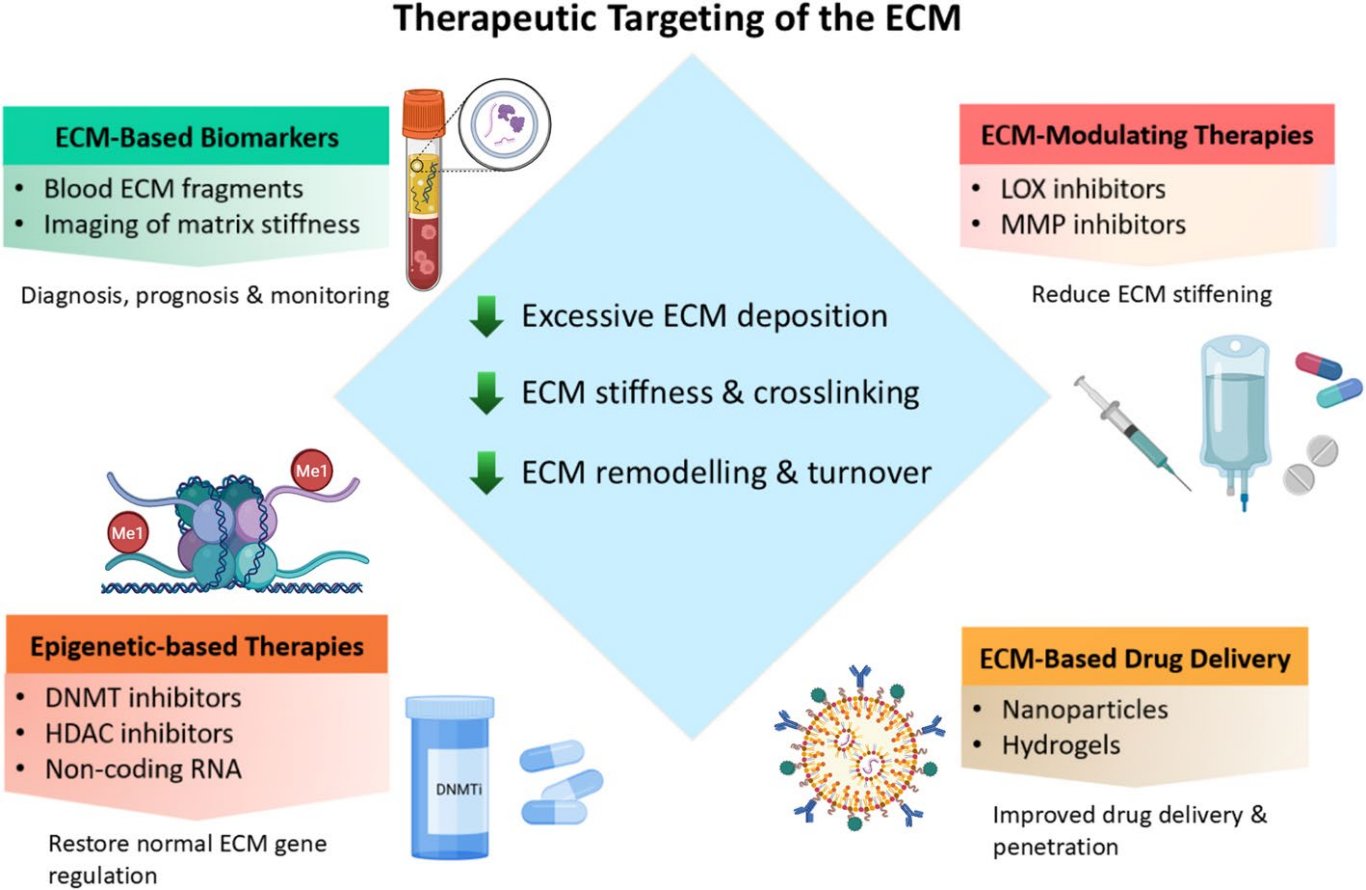
Schematic illustration of integrated therapeutic strategies targeting ECM dysregulation to restore ECM homeostasis. The central node defines the therapeutic objective: attenuation of excessive ECM deposition, crosslinking, stiffness, and aberrant turnover, with normalisation of mechanotransduction signalling. (Top left) ECM-based biomarkers capture matrix-derived fragments and stiffness-associated signatures, enabling early detection, prognosis, and disease monitoring. (Top right) Direct ECM-modulating therapies (e.g., LOX inhibitors, MMP inhibitors to target matrix crosslinking, cell–matrix adhesion, and proteolytic remodelling to reduce pathological stiffening and scarring. (Bottom left) Epigenetic-based interventions reprogram transcriptional circuits governing ECM synthesis and remodelling, thereby suppressing fibrogenic activation and restoring matrix gene regulation. (Bottom right) ECM-guided drug delivery platforms exploit matrix composition and mechanics to enhance tissue-specific accumulation, penetration, and controlled release. Created in BioRender.com

Therapeutic strategies that directly target the ECM primarily aim to reduce pathological stiffness, inhibit excessive remodelling, or disrupt mechanotransduction pathways that convert mechanical cues into pro-fibrotic and pro-tumour signalling. The crosstalk between the ECM in TME and epigenetic regulation creates powerful reinforcing feedback loops that entrench treatment resistance. Beyond serving as a therapeutic target, the ECM increasingly functions as a source of clinically informative biomarkers, a blueprint for advanced biomaterial design, and a versatile platform for drug delivery (Fig. 5). Dynamic ECM remodelling during disease progression generates bioactive fragments, altered crosslinking patterns, and changes in stiffness that reflect pathological states and therapeutic response. Targeting these epigenetic regulators offers a mechanistically precise method to reverse pathogenic ECM accumulation across multiple diseases; rather than addressing individual components in isolation, it is crucial for overcoming the root of certain resistance mechanisms and for supporting improvements in drug delivery and immune cell infiltration.

### Preclinical strategies

ECM-derived molecules and remodelling signatures are emerging as robust biomarkers for disease diagnosis, prognosis, and treatment monitoring. Modulating ECM stiffness and degrading its components are promising approaches to enhance drug penetration and immune cell access. Proteolytic degradation of collagens, elastin, and proteoglycans releases circulating ECM fragments (matrikines) that reflect tissue-specific remodelling activity (Table 3). Preclinical studies consistently demonstrate that suppressing LOX activity is able to normalise tissue stiffness by reducing collagen crosslinking, promoting immune cell infiltration, and improving drug penetration in both fibrosis and cancer models [59]. In cancer and fibrosis, elevated levels of collagen-derived neo-epitopes, LOX activity, and MMP profiles correlate with disease stage, metastatic potential, and patient outcomes [288–290]. Similarly, increased ECM stiffness and crosslinking have been associated with poor prognosis in breast, lung, and liver cancers, as well as progressive fibrosis and cardiovascular disease [291, 292]. In addition, normalising the ECM architecture, for instance, by reducing collagen crosslinking, can improve T cell migration and boost the effectiveness of anti-PD-1 therapies [248]. These strategies aim to dismantle the physical and signalling barriers imposed by the aberrant ECM, thereby "normalising" the TME to make it more permissive to therapeutic agents and immune responses.

**Table 3:** Therapeutic strategies for the ECM from preclinical and clinical perspectives

Therapeutic Strategy	Mechanism/Key Findings	Disease Context	Outcome/Implications	Refs.
LOX/Collagen Crosslinking Inhibition	Reduces collagen crosslinking; normalises tissue stiffness; improves immune infiltration and drug penetration	Fibrosis, cancer	Enhances T cell migration; improves response to anti-PD-1	[59, 248]
Targeting Collagen Deposition & Remodelling	High collagen elevates interstitial pressure and drug resistance; COL11A1 activates SMAD signalling and cisplatin resistance	Solid tumours; NSCLC	Addresses CAF-mediated resistance; improves drug delivery	[249–251]
MMP Inhibition	Blocks ECM degradation/remodelling; regulates immune infiltration	Cancer; fibrosis	Limits tumour growth/metastasis; enhances immunotherapy efficacy	[248, 249, 252, 253],
DNMTi	Demethylates promoters; reactivates TSGs; suppresses fibroblast activation and collagen synthesis	Hematologic cancers; solid tumours; lung/liver/cardiac fibrosis	Anti-fibrotic effects; context-dependent responses; may transiently promote invasion	[254–262]
HDAC inhibitors	Increase histone acetylation; suppress TGF-β–induced fibroblast activation; reduce α-SMA	Lung, cardiac fibrosis; cancer	Normalises collagen turnover; modulates MMP/TIMP balance	[263–265]
HMT Inhibitors	Block H3K27me3-mediated repression of anti-fibrotic genes; inhibit EZH2	Fibrosis; cancer	Reduce ECM stiffness; reverse fibrosis; tazemetostat shows clinical relevance	[266–268]
miRNA-Based Therapies	miR-29 mimics reduce collagen I/III; anti-miR-21 reduces fibroblast activation	Lung/liver fibrosis; cardiac/renal fibrosis	Strong anti-fibrotic effects; suppress ECM deposition	[100, 269]
lncRNA Antagonists	Target H19, MALAT1, HOTAIR to reverse repression of anti-fibrotic genes	Fibrosis; cancer	Reduce ECM stiffening and scarring	[270]
circRNA Modulation	Inhibition of circHIPK3 reduces collagen synthesis and fibrosis	Liver fibrosis	Anti-fibrotic effects	[271, 272]
Epigenetic-ECM Combination Therapies	Dual targeting of ECM stiffness and epigenetic regulators enhances therapeutic sensitivity	Cancer; fibrosis	Reprograms CAFs; decreases matrix stiffening; improves drug response	[264, 273–276]
Immuno-Epigenetic Combination (DNMTi + ICIs)	DNMTi enhance tumour antigenicity, MHC I, CTL activity	Solid tumours, melanoma	Converts “cold” tumours to “hot”; promising early-phase trials	[143, 277–283]
Anti-Fibrotic Small Molecules (Pirfenidone, Nintedanib)	Reduce fibroblast activation and collagen deposition; alter mechanical microenvironment	Idiopathic pulmonary fibrosis	Improve ECM-related outcomes without altering epigenetic marks	[284, 285]
Integrin-FAK Inhibition	Blocks mechanotransduction (integrin-FAK-RhoA/YAP)	Cancer; fibrosis	Reduces stiffness-driven signalling in multiple clinical trials	[286, 287]

Enhanced collagen deposition and remodelling result in elevated interstitial pressure, which impairs drug delivery efficacy [249]. In the TME, distinct drug resistance patterns are associated with collagen fibres, particularly collagen type I [249]. Chemotherapy resistance is often associated with increased tissue stiffness, driven by collagen cross-linking, with specific collagen types playing critical roles [250, 251]. Notably, COL11A1 has been linked to cisplatin resistance in NSCLC by activating the SMAD signalling pathway [249]. Consequently, it underscores the need for targeted strategies that address CAFs and the ECM to improve therapeutic outcomes. Another strategy is to block enzymes involved in ECM remodelling, such as MMPs, to prevent the ECM from aiding in tumour growth and metastasis [259], and fibrosis [252, 253], as MMPs can cleave nearly all ECM components and facilitate ECM remodelling [249], immune cell infiltration and enhancing the efficacy of immunotherapies [248]. However, the specific effects of MMP methylation can be complex and vary depending on the MMP, the methylation site, and the tumour context.

Epigenetic therapeutics, particularly DNMTi, represent a major class of ECM-modifying interventions. Agents such as azacitidine (5-aza) and decitabine (5-aza-dC) are widely used in epigenetic studies, where they demethylate gene promoters and reactivate silenced tumour suppressor genes. Although clinically approved for hematologic malignancies, DNMTi are increasingly explored in solid tumours with promising preclinical outcomes. Inhibition of DNMT1 and DNMT3A has been shown to suppress fibroblast activation and reduce collagen synthesis in models of lung, liver, and cardiac fibrosis [254–256], meanwhile, targeting DNMT3B with siRNA or 5-aza-dC significantly attenuates cardiac fibrosis [257]. Blocking aberrant DNMT activity can also restore anti-fibrotic gene expression and reverse pathological ECM deposition in fibrosis and cancer models [258]. While many studies report anti-fibrotic effects of 5-aza-dC across organs [259], existing studies also describe pro-fibrotic responses in lung and cardiac fibroblasts [260, 261], reflecting its context-dependent actions. Because ECM-related genes are epigenetically regulated, DNMTi can reshape matrix-associated transcriptional programs, and in certain settings may transiently enhance tumour invasion, necessitating careful preclinical and clinical assessment [262]. Collectively, these findings highlight the complexity of DNMT-mediated regulation of ECM remodelling and the need for deeper investigation across different organ systems.

Histone modifications regulate chromatin structure and gene expression. By targeting the enzymes that add or remove these modifications, researchers can influence the transcription of key genes involved in ECM remodelling. Studies on the use of HDAC inhibitors, such as panobinostat, vorinostat, and trichostatin A, have successfully normalised collagen turnover in lung and cardiac fibrosis models by downregulating α-SMA expression and blocking TGF-β-induced fibroblast activation [263–265]. These agents block histone deacetylases, increasing acetylation levels and altering the expression of ECM-related genes. Meanwhile, histone methylation can either activate or repress gene expression depending on the specific residue and the degree of methylation. The use of histone methyltransferase (HMT) inhibitors is reported to reduce ECM stiffness and reverse the fibrosis process by preventing H3K27me3-mediated silencing of anti-fibrotic genes [266, 267]. In addition, HMT inhibitors, particularly those targeting EZH2, can modulate ECM-regulating gene networks. Tazemetostat, an EZH2 inhibitor approved for certain cancers, highlights emerging opportunities for targeting H3K27me3-mediated gene silencing within ECM-remodelling pathways [267, 268].

Non-coding RNA-based therapies have demonstrated substantial pre-clinical potential in targeting ECM dysregulation across fibrotic and malignant diseases. Therapeutic miRNA mimics, such as miR-29, effectively reduce the production of collagen I and III and have demonstrated anti-fibrotic activity in models of lung and liver fibrosis [269]. Conversely, miRNA inhibitors such as anti-miR-21 suppress fibroblast activation, attenuate inflammatory signalling, and significantly reduce cardiac and renal fibrosis [100]. Targeting pathogenic lncRNAs, including H19, MALAT1, and HOTAIR, has been shown to reverse epigenetic silencing of anti-fibrotic genes, thereby diminishing ECM stiffening and tissue scarring [270]. Additionally, modulation of circular RNAs, particularly by inhibiting pro-fibrotic circHIPK3, reduces collagen synthesis and ameliorates fibrosis in liver disease models [271, 272]. Collectively, these findings highlight ncRNA-directed therapies as promising strategies for restoring ECM homeostasis and mitigating epigenetically driven tissue pathology.

### Current clinical trials and translational challenges

The therapeutic targeting of ECM-epigenetic interactions is an emerging field with significant implications for various diseases, particularly cancer and fibrotic conditions. Current therapeutic strategies targeting the dysregulated ECM in pathological conditions focus on inhibiting matrix degradation to prevent metastasis and on facilitating matrix degradation to enhance drug and immune cell penetration. However, these approaches can lead to unintended consequences such as tumour growth promotion and drug resistance [293, 294]. Several HDAC inhibitors are currently in clinical testing for cancer and fibrotic diseases, reflecting broad evidence of anti-fibrotic and anti-tumour effects in preclinical models [264, 273]. In neuroblastoma, epigenetic modulators such as HDAC inhibitors and DNMTi are being investigated for their potential to improve outcomes in high-risk patients [274]. Similarly, in systemic sclerosis and cardiac fibrosis, targeting epigenetic mechanisms such as DNA methylation and histone modification is being explored to mitigate disease progression [274–276]. Additionally, an ECM-directed interventions include anti-fibrotic small molecules, such as pirfenidone and nintedanib, which are approved for the treatment of idiopathic pulmonary fibrosis and effectively reduce fibroblast activation and collagen deposition [284, 285]. These drugs do not directly reverse epigenetic marks but modulate the mechanical microenvironment and downstream signalling pathways that influence epigenetic states [284, 285].

Combination therapies are currently receiving greater attention as alternatives to address the complex bidirectional relationship between ECM and epigenetic factors in various diseases, aiming to achieve more effective outcomes despite using individual interventions. A combination of epigenetic agents with immunotherapy may help overcome ECM-driven immune exclusion in solid tumours [126]. Early-phase trials and translational studies support the potential of these combined modalities to overcome matrix-mediated therapeutic resistance and restore a more permissive microenvironment for treatment efficacy [286, 295]. Preclinical studies demonstrate that ECM-softening approaches, including LOX inhibition or collagen deposition blockade, can increase sensitivity to epigenetic drugs. Conversely, DNMTi, HDACi, or EZH2 inhibitors can reprogram cancer-associated fibroblasts and tumour cells, reducing matrix remodelling and stiffening. These reciprocal effects provide a strong rationale for dual-targeting strategies and offer novel insights into therapeutic approaches involving the epigenetic-ECM relationship.

Current clinical trials are also investigating the use of DNMTi in combination with conventional and immune-based therapies. As DNA methylation regulates cellular proliferation, differentiation, migration, and immune activation [277, 278], targeting aberrant methylation patterns may overcome immune evasion mechanisms [278, 279]. DNMTi can enhance tumour antigen presentation, upregulate MHC I expression, and increase cytotoxic T lymphocyte (CTL) function by inducing pro-inflammatory cytokines and chemokines [280]. Their use alongside immune checkpoint inhibitors (ICIs) aims to transform immunologically “cold” tumours into “hot” ones, improving immunotherapy outcomes [281, 282]. Encouragingly, the NIBIT-M4 trial demonstrated the safety and immunomodulatory potential of combining hypomethylating agents with ICIs in melanoma patients [283]. While most studies remain in early clinical phases [81, 143, 296–299], these combination strategies hold strong promise for overcoming resistance and enhancing therapeutic efficacy. Early-phase trials and translational studies support the potential of these combined modalities to overcome matrix-mediated therapeutic resistance and restore a more permissive microenvironment for treatment efficacy [286, 295].

Despite encouraging mechanistic data, clinical translation has been difficult. The multifunctional nature of the ECM and its role in various stages of tumour development, including initiation, progression, and chemoresistance, pose significant challenges in translating laboratory findings into clinical applications. This is because the multifunctional nature of ECM components complicates the identification of specific therapeutic targets [275, 300]. For example, simtuzumab, an anti-LOXL2 monoclonal antibody, failed in Phase II trials for fibrosis, although next-generation LOX/LOXL inhibitors and small molecules remain under active development [295, 301]. Parallel efforts focus on inhibiting integrin-FAK mechanotransduction, a central pathway through which cells sense ECM stiffness, such as defactinib and several integrin antagonists that block the connection of integrin-FAK-RhoA/YAP cascade and have progressed into clinical trials for cancer, with ongoing evaluation in combination regimens [286, 287]. The lack of matrix-targeting agents in mainstream clinical use highlights the need for better integration of new technologies and a deeper understanding of the complex roles of the ECM [294, 302].

Effective delivery and transport of therapeutics within the TME and ECM are major hurdles, as the ECM's physical and biochemical barriers can limit their penetration and efficacy [303]. Additionally, ensuring the specificity of ECM-targeted therapies to avoid off-target effects and minimise side effects remains a critical challenge [294]. Translating preclinical findings to clinical practice is fraught with challenges, including variability in patient responses, the complexity of human diseases, and the need for robust biomarkers to predict treatment outcomes. Primarily, many current models do not adequately replicate the human ECM environment, hindering the prediction of clinical outcomes. This gap underscores the need for more robust translational models that better replicate the complexities of human diseases [302]. For instance, the translation of anti-fibrotic therapies from the laboratory to clinical trials has been limited, underscoring the need for more rigorous clinical studies and combination therapies [276, 304].

To date, there remains limited preclinical research addressing their impact on ECM regulation, with epigenetic-based therapies approved in clinical trials being hampered by the dynamic nature of epigenetic regulation, which adds another layer of complexity (Fig. 5). The interplay between ECM components and epigenetic mechanisms can influence disease progression and therapeutic responses, necessitating a comprehensive understanding of these interactions [274, 275]. For instance, despite these promising preclinical findings, the clinical translation of DNMTi targeting ECM remodelling enzymes remains limited due to concerns regarding achieving sufficient specificity without disrupting normal ECM integrity [305], systemic toxicity [306], potential off-target effects [307] and effective dosing maintenance. Continued efforts to refine these therapies are essential to selectively reverse hypermethylation without triggering global hypomethylation and to promote the activation of genes associated with fibrotic or metastatic processes.

## Future directions and therapeutic opportunities at the ECM-epigenetics-disease nexus

### Single-cell ECM omics and spatial profiling

Advances in single-cell ECM omics and spatial profiling have substantially deepened the understanding of how the ECM shapes tissue microenvironments in both normal and diseased conditions. Single-cell ECM omics integrates high-resolution single-cell technologies with ECM analysis to characterise ECM composition, biophysical properties, and cell–ECM interactions at the level of individual cells [308]. This approach helps clarify how various cell types respond to mechanical and biochemical cues during tissue development, homeostasis, and pathological progression [308]. Technologies such as single-cell RNA sequencing, spatial transcriptomics, and single-cell proteomics have been instrumental in defining the heterogeneity of ECM-producing and ECM-responsive cells. These tools uncover rare but biologically significant cell populations, reveal cell-state transitions that are masked in bulk analyses, and enable the detailed mapping of interactions relevant to metastasis, immune regulation, and fibrosis [309]. For example, single-cell studies have elucidated metastatic cell–cell signalling networks [309], epithelial-stromal interactions that regulate ECM turnover [310], and the differential responses of cell subsets to ECM stiffness [311]. Moreover, single-cell multimodal omics now provide the ability to simultaneously capture DNA methylation, chromatin accessibility, and transcriptomic activity, offering a more complete view of ECM-associated regulatory mechanisms at the cellular level [308].

Spatial profiling technologies extend these insights by preserving tissue architecture and allowing gene expression and protein localisation to be mapped in situ. This spatial context is crucial for understanding how cells interact with neighbouring cells and with their ECM, influencing structural organisation and functional outcomes in tissues [274, 312]. Spatial transcriptomics has revealed localised inflammatory niches and pathological microenvironments in diseases such as atopic dermatitis and chronic obstructive pulmonary disease, identifying new biomarkers and therapeutic targets [274, 275]. Technological innovations, such as Extracellular Protein Identification Cytometry (EPIC), further support the high-throughput, single-cell-level measurement of ECM protein deposition, enabling the precise quantification of matrix production heterogeneity among individual cells [293]. The integration of single-cell and spatial omics enables comprehensive tissue profiling, linking molecular signatures to specific anatomical contexts. Advances in machine learning and computational modelling facilitate the interpretation of large, complex datasets generated by these approaches [276, 302]. These developments collectively support the emerging applications of single-cell ECM profiling in personalised medicine and regenerative medicine, including the tailoring of interventions based on patient-specific microenvironmental signatures [303, 312] and the design of biomaterials that mimic natural ECM cues to optimise tissue repair [300, 313].

### Personalised ECM-targeted therapies

The integration of ECM biology with epigenetic insights is paving the way for increasingly personalised therapeutic strategies. Future ECM-targeted therapies are expected to incorporate advanced delivery platforms, such as nanoparticles, hydrogels, and engineered biomaterials, to enhance drug specificity, reduce systemic toxicity, and improve therapeutic penetration into dense ECM-rich tissues [293, 300]. Epigenetic therapies, including DNMTi, HDAC inhibitors, and chromatin-modifying agents, offer promising avenues for reprogramming aberrant ECM production, mitigating fibrosis, and normalising tumour microenvironments [274, 275, 312]. The combination of ECM-directed interventions with epigenetic modulators has the potential to overcome the limitations of single-pathway targeting by addressing both structural and regulatory components of diseased tissues. Personalised medicine approaches are also expanding with patient-specific genomic, epigenomic, and ECM-derived biomarkers, enabling stratification of individuals based on predicted therapeutic responsiveness and risk profiles [314, 315]. However, these advances also highlight the need for robust ethical and regulatory frameworks, particularly regarding patient consent, long-term monitoring, and safety evaluation of new ECM-epigenetic-targeted interventions [315].

Understanding ECM-epigenetic interactions also enhances the discovery of biomarkers, as ECM stiffness and biochemical composition influence critical cellular behaviours, including growth, differentiation, migration, and apoptosis. In cancer, increased matrix stiffness drives tumour progression by altering mechanotransduction pathways and promoting invasive phenotypes [303, 313]. Identifying ECM-based biomarkers and epigenetic signatures linked to disease progression supports earlier detection and more precise therapeutic targeting [304, 314]. Furthermore, innovative delivery systems, such as ECM-targeted nanoparticles, show promise for improving drug localisation and enhancing imaging specificity in preclinical tumour models [276, 300]. Advances in proteomics and imaging-based mechanobiology now enable quantitative assessment of ECM composition and biomechanics, supporting the use of ECM signatures as non-invasive biomarkers and predictive indicators of therapeutic responsiveness [16, 57]. Together, these approaches illustrate the potential of personalised ECM-targeted therapy as a major direction for future translational research.

### Challenges and future directions

The biological complexity of ECM-epigenetic interplay also presents translational challenges. The ECM operates through redundant, compensatory pathways, meaning that inhibiting a single molecule may not yield the desired therapeutic effect. The complexities underscore the importance of optimising drug combinations, dosing schedules, and treatment sequencing to safely modulate ECM structure while preventing excessive matrix degradation or tumour spread. Despite rapid progress, several scientific and translational challenges remain. A major obstacle is the tumour heterogeneity that exists within individual tumours and between different patients, resulting in diverse therapeutic responses across sub-clonal populations [6, 122]. Epigenetic plasticity further amplifies this challenge by enabling cancer cells to dynamically adapt to therapeutic pressure, contributing to drug resistance and treatment failure [316]. The development of personalised ECM-epigenetic therapies requires integrating multi-omics datasets, including DNA methylation, chromatin accessibility, transcriptomics, proteomics, and spatial information. However, such integration requires advanced computational pipelines, significant data-processing capacities, and methodological standardisation to ensure reproducibility [307].

Looking forward, technological innovation offers potential solutions as insights into ECM composition, architecture, and mechanics have directly informed the design of advanced biomaterials for tissue engineering and regenerative medicine. Biomimetic scaffolds incorporating ECM-derived components such as collagen, laminin, hyaluronic acid, and decellularised ECM can recapitulate native biochemical and mechanical cues, thereby guiding cell adhesion, differentiation, and tissue regeneration [28, 317, 318]. Adjustable matrix stiffness, fibre alignment, and ligand presentation enable precise control of cell fate decisions, making ECM-inspired materials valuable tools for modelling disease, testing therapeutics, and repairing damaged tissues. Nanoparticle-based delivery systems, ECM-targeted biomaterials, and spatially resolved epigenomic profiling are emerging approaches that may overcome limitations in drug delivery, tumour penetration, and cellular specificity [276, 300]. ECM-based hydrogels and injectable matrices allow controlled, sustained release of therapeutic agents while maintaining localised drug concentrations and reducing systemic toxicity [319]. In oncology, ECM-targeted nanoparticles exploit overexpressed matrix components such as fibronectin, collagen, or hyaluronan to enhance tumour-specific accumulation and penetration [320]. Enzyme-responsive delivery systems that release drugs in response to MMP activity or altered ECM stiffness further improve spatial precision in diseased tissues [321]. Further identification of ECM-associated and epigenetic biomarkers promises earlier disease detection and more effective therapeutic stratification [304, 314]. Continued efforts to standardise single-cell and spatial omics methodologies, improve data reproducibility, and refine computational tools will be essential for translating laboratory discoveries into clinical applications [302, 312]. Importantly, integrating epigenetic and mechanotransduction insights into biomaterial design has revealed that ECM-mimetic substrates can modulate chromatin organisation and transcriptional programs, further enhancing regenerative outcomes [322]. Advancing the understanding of ECM-epigenetic crosstalk will be crucial for developing next-generation therapies that effectively target the structural and regulatory components of diverse diseases, particularly cancer and fibrotic disorders.

## Conclusion

The ECM is increasingly recognised as a dynamic regulatory platform that integrates biochemical signalling with mechanical forces to maintain tissue homeostasis. Through tightly regulated synthesis, degradation, crosslinking, and structural organisation, the ECM governs mechanotransduction and cellular behaviour, therefore essential for preserving physiological balance. Disruption of this tightly controlled network transforms the ECM into a driver of pathology, where altered stiffness, composition, and spatial organisation become distinguishing hallmarks of diseased tissues. Thus, aberrant ECM remodelling is not merely a consequence of disease but a central determinant of disease initiation, progression, and therapeutic response.

A key insight from this review is the bidirectional, self-sustaining interplay between ECM remodelling and epigenetic regulation that shapes tissue homeostasis, disease progression, and therapeutic response across diseases. ECM stiffness, composition, and mechanical forces can reprogram cellular epigenomes, driving fibroblast activation, EMT, immune evasion, and tumour aggressiveness. Conversely, the epigenetic mechanisms govern ECM synthesis, degradation, crosslinking, and mechanotransduction, forming self-reinforcing loops that sustain pathological remodelling, fibrosis, organ dysfunction, and treatment resistance. Understanding this complex crosstalk is the key to managing the diseases and possibly restoring the normal pathophysiology. The aberrant regulation of ECM remodelling has emerged as the main culprit in various diseases and is often linked to epigenetic mechanisms. However, as both ECM and epigenetics are complex, a thorough understanding of their link is required to facilitate management and therapeutic strategies.

Therapeutically, this integrated view expands the scope of intervention beyond simple matrix inhibition. ECM components can serve simultaneously as biomarkers for early detection, indicators of disease stratification, and platforms for targeted drug delivery. The convergence of ECM-based diagnostics with epigenetic therapies offers a particularly promising opportunity, enabling spatially informed and mechanistically precise interventions. In our analysis, the most impactful future strategies will likely combine matrix-normalising approaches with epigenetic modulators to disrupt pathological feedback loops rather than targeting single nodes in isolation. Such combinatorial frameworks may enhance treatment durability and reduce relapse driven by microenvironmental adaptation.

Therapeutically, this systems-level perspective supports strategies that extend beyond direct matrix inhibition. ECM components offer opportunities as biomarkers, stratification tools, and targeted delivery platforms, while epigenetic drugs and RNA-based agents provide precision in reprogramming aberrant transcriptional states. Nevertheless, clinical translation remains challenging due to pathway redundancy, compensatory remodelling, organ-specific biomechanics, off-target effects, and delivery barriers. Combination strategies that jointly target ECM mechanics and epigenetic drivers are emerging as an effective approach to disrupt these pathogenic loops and improve treatment sensitivity. Future progress will depend on integrating single-cell omics, spatial epigenomics, and mechanobiology to map patient-specific ECM and epigenetic states, paving the way for more durable, targeted, and personalised interventions.

## Data Availability

Not applicable.
